# Integrated metabolomic and transcriptomic analysis of *Camellia* sinensis var. *pubilimba* ‘Rucheng Baimaocha’ reveals distinct flavonoid and strictinin biosynthesis

**DOI:** 10.1186/s12870-026-09521-7

**Published:** 2026-07-15

**Authors:** Furong Qu, Yang Zhao, Peidi Yang, Yang Cheng, Zhen Liu

**Affiliations:** 1 Tea Research Institute, Hunan Academy of Agricultural Sciences, Changsha, 410125 China; 2 Yuelushan Laboratory, Changsha, 410128 China

**Keywords:** Tea plant, Rucheng Baimaocha, Metabolomic, Transcriptomic, Flavonoid, Strictinin

## Abstract

**Background:**

‘Rucheng Baimaocha’ (RCBMC) is a traditional tea landrace found in Hunan Province, China. Its morphological characteristics differ substantially from those of the other cultivars, featuring larger mature leaves with thicker cuticle layers. Both young buds and leaf undersides are densely covered in silvery-white trichomes, indicating that RCBMC has a distinct metabolite composition compared to other cultivars. To elucidate the metabolic profile differences and their underlying molecular mechanisms, we conducted an integrated metabolomic and transcriptomic analysis of RCBMC and representative cultivars.

**Results:**

Metabolomics revealed that RCBMC accumulated higher levels of non-epicatechins, such as catechin, catechin gallate, and gallocatechin gallate, and the ellagitannin strictinin, whereas flavonoid glycosides were significantly lower. Transcriptomics identified 11,775 differentially expressed genes with key shifts in the flavonoid pathway: upregulated *LAR* and downregulated *ANS* gene expression collectively redirected metabolic flux toward non-epicatechin synthesis. The downregulation of multiple *UGT* genes was correlated with reduced flavonoid glycoside levels. Weighted gene co-expression network analysis further identified transcription factors strongly associated with metabolite accumulation. Quantitative analysis of 607 medium- and small-leaf tea germplasms indicated that strictinin content was genetically influenced and seasonally regulated, with RCBMC exhibiting notably high levels. Correlation analysis identified candidate genes from the *SCPL*, *CXE*, and *LAC* families that are potentially involved in strictinin biosynthesis.

**Conclusions:**

This study revealed a unique pattern of bioactive compound accumulation in RCBMC and provides valuable germplasm resources and genetic targets for breeding tea cultivars with enhanced functional components.

**Supplementary Information:**

The online version contains supplementary material available at https://doi.org/10.1186/s12870-026-09521-7.

## Introduction

Tea plant (*Camellia sinensis* (L.) Kuntze) is a globally important leaf-based cash crop valued for its distinctive sensory qualities and health attributes, primarily attributed to the accumulation of various secondary metabolites, including tea polyphenols, catechins, and caffeine, in its leaves [1, 2]. The global consumer market is increasingly demanding high-quality teas with diverse functional properties. The development of innovative tea products relies on a wide variety of tea germplasm resources.

Extensively distributed tea landraces contain substantial genetic variation, making them crucial resources for identifying distinctive quality traits and broadening the genetic background for breeding [3–7]. Currently, tea production relies primarily on limited, widely cultivated superior cultivars. However, numerous landraces with potentially exceptional qualities remain to be systematically investigated.

‘Rucheng Baimaocha’ (RCBMC) originates in the mountainous Jiulongjiang region. It is a landrace in Hunan Province, China, and is a typical representative of *Camellia sinensis* var. *pubilimba*. RCBMC exhibits the highest genetic differentiation coefficient among the four typical Hunan tea landraces, indicating that this germplasm resource exhibits considerable genetic distinctiveness [8]. Compared with cultivated varieties that have undergone long-term artificial selection and exhibit stable traits, these germplasm resources typically retain richer genetic diversity and may correspond to unique metabolite compositions. Therefore, RCBMC is a potentially high-value breeding material. Current studies on the germplasm resources of RCBMC have primarily focused on morphological trait analysis [9], tea quality evaluation [10–12], and the establishment of core germplasm collections [13]. However, the specific differences between RCBMC and cultivars remain unclear. These differences extend beyond the composition of metabolic products and include the key gene expression patterns that drive these phenotypic variations. Addressing this question will enhance our understanding of this distinctive resource and promote its innovative utilization.

Within the complex metabolome of tea plants, flavonoids determine the flavor and bioactivity of tea leaves. In addition, certain specific metabolites often confer specialized functional properties and developmental value on tea plant germplasm resources. Strictinin, a type of ellagitannin, has garnered increasing attention for its effects in reducing uric acid [14], anti-obesity [15], and blood glucose-lowering properties [16]. In common cultivated tea varieties, high accumulation of strictinin is relatively rare, and its biosynthetic pathway within tea plants remains unclear. Identifying tea germplasm resources with high strictinin content and studying their biosynthetic pathways is of great significance for the breeding of functional tea varieties.

Recently, integrated metabolomic and transcriptomic analyses have been widely applied in the study of tea germplasm resources [17–19]. In this study, we employed metabolomics to analyze the metabolite profiles of seven cultivars and three individual plants from the RCBMC population. We systematically elucidated the unique metabolic characteristics of RCBMC, particularly focusing on the flavonoid composition and strictinin content. Transcriptomics identified differentially expressed genes between RCBMC and cultivars across the entire genome, whereas association analysis revealed key genes involved in the biosynthesis and regulation of differentially expressed metabolites, notably those related to flavonoid and strictinin biosynthesis. Additionally, we quantified the strictinin content in 607 small- and medium-leaf tea germplasm resources and performed multi-seasonal testing on representative samples to characterize its distribution pattern, genetic dependence, and seasonal variation.

This study aimed to (1) evaluate the resource value of RCBMC, thereby providing a scientific basis for its development and utilization. and (2) provide germplasm resources and candidate genes for the genetic improvement of tea plant quality and the targeted enhancement of flavonoids and strictinin.

## Materials and methods

### Chemicals

Methanol (chromatographic grade) and acetonitrile (chromatographic grade) were purchased from Merck (USA). Formic acid was purchased from TCI (Japan). Catechin (C) (purity ≥ 98%), gallocatechin (GC) (purity ≥ 98%), epicatechin (EC) (purity ≥ 98%), epigallocatechin (EGC) (purity ≥ 98%), epicatechin gallate (ECG) (purity ≥ 98%), gallocatechin gallate (GCG) (purity ≥ 98%), epigallocatechin gallate (EGCG) (purity ≥ 98%), catechin gallate (CG) (purity ≥ 98%) reference standards were purchased from Sigma (USA). Strictinin (purity ≥ 95%) reference standard was purchased from Yuanye (China).

### Plant materials

All tea germplasm resources used in the study coming from the China National Small and Medium-leaf Tea Germplasm Resource Nursery (Changsha) and were cultivated and managed under identical environmental conditions.

This study selected three individuals from the ‘Rucheng Baimaocha’ (RCBMC) population and seven nationally recognized tea varieties as experimental materials. These seven tea varieties are native to different provinces in China and are suitable for producing different types of tea. They are: *Camellia sinensis* ‘Zhuyeqi’ (Hunan Province; green and black tea), *Camellia sinensis* ‘Fuding Dabaicha’ (Fujian Province; green, black, and white tea), *Camellia sinensis* ‘Jinguanyin’ (Fujian Province; oolong tea), *Camellia sinensis* ‘Longjing 43’ and *Camellia sinensis* ‘Zhongcha 108’ (Zhejiang Province; green tea), *Camellia sinensis* ‘Anhui 1’ (Anhui Province; green and black tea), *Camellia sinensis* ‘Echa 1’ (Hubei Province; green tea).

On April 15, 2024, two and a bud were harvested from each tea plant, immediately frozen in liquid nitrogen, and stored at − 80 ℃ for transcriptomic analysis. Leaves were freeze-dried for metabolomic analyses. Seven cultivated varieties were used as the control (CK) group, and three RCBMC individuals were used as the RCBMC group.

Furthermore, for the large-scale population screening of strictinin content, from April 3 to April 27, 2024, two and a bud were collected from a diverse panel of 607 medium- and small-leaf tea plants (including 25 RCBMC individuals). The 607 germplasm resources were all sourced from the China National Small and Medium-leaf Tea Germplasm Resource Nursery (Changsha). The screening criterion was designed to encompass the maximum available genetic diversity within this repository, thereby providing an unbiased dataset for evaluating the relative strictinin content within the broader tea population.

To evaluate seasonal variations, additional sampling of the RCBMC and CK individuals was conducted on September 20, 2024, and April 14, 2025 following the same protocols.

### Tea sample preparation

Powder (0.1 g) was accurately weighed into a 50 mL centrifuge tube. Subsequently, 20 mL of 70% methanol solution was added, shaken thoroughly, and placed in a 70 °C water bath for 30 min. The tubes were shaken every 10 min to enhance metabolite extraction efficiency. The mixture was cooled to room temperature and centrifuged at 8000 rpm for 10 min. The supernatant was transferred through a 0.45 μm filter membrane, and 1.5 mL of the filtrate was dispensed into an injection vial. Three parallel replicates were prepared for each sample for subsequent analysis.

### Non-targeted metabolomics detection and analysis

An ultra-high-performance liquid chromatography-quadrupole Orbitrap mass spectrometer (UHPLC-Q-Exactive/MS) was purchased from Thermo Fisher Scientific Inc. (USA).

UHPLC was performed with a T3 column (100 mm × 2.1 mm, 1.7 μm, Waters, UK) at 40 °C. The flow rate was set to 0.4 mL/min, and the injection volume was 3 µL. The mobile phases comprised Phase A (0.1% formic acid solution) and Phase B (acetonitrile solution containing 0.1% formic acid). The elution program was as follows: 0 min, 2% Phase B; 0.5 min, 2% Phase B; 8 min, 15% Phase B; 13 min, 35% B; 15 min, 70% B; 16 min, 85% B; 16.5 min, 2% B; and 20 min, 2% B.

Quadrupole Orbitrap Mass Spectrometry was conducted using an electron spray ionization (ESI) source. The detection occurred by scanning simultaneously in both positive and negative ion modes. The capillary voltage was set at 3.5 kV, with a capillary temperature of 300 °C. The auxiliary gas temperature was controlled at 350 °C with a flow rate of 10 L/min. Mass spectrometry analysis was performed over a mass-to-charge ratio (m/z) scanning range of 80–1200.

Raw spectra obtained from the UHPLC-Q-Exactive/MS analysis were processed using Compound Discoverer 3.3 software for peak matching and peak area extraction. The peak tables generated using this method were imported into Microsoft Excel for subsequent statistical analyses.

### High-performance liquid chromatography (HPLC) detection

Quantitative determination of the catechin and strictinin components was performed using an LC-20AD high-performance liquid chromatograph (Shimadzu, Japan).

HPLC was performed using a Waters XSelect HSS T3 column (4.6 mm × 250 mm, 5 μm, U.K.) at 41 °C. The detection wavelength was set to 280 nm, with an injection volume of 10 µL and a flow rate of 1 mL/min. Mobile phase A consisted of 0.1% (v/v) formic acid, while mobile phase B was acetonitrile. The gradient elution conditions were as follows: 0 min, 3% B; 13 min, 6% B; 17 min, 6% B; 26.5 min, 6.4% B; 30 min, 6.4% B; 35 min, 11% B; 40 min, 13% B; 50.00 min, 40% B; 53 min, 40% B; 56 min, 3% B; and 65 min, 3% B.

Calibration curves generated from standard solutions were used to perform an absolute quantitative analysis of the metabolites detected by HPLC.

### RNA-Seq and analysis

Total RNAs were extracted following the manufacturer’s protocol using the EASY-spin Plus Complex Plant RNA kit (Aidlab Biotechnologies Company, China) and quantified using a NanoDrop (Thermo Fisher Scientific, USA). Transcriptome sequencing was performed by Novogene Co., Ltd., using the Illumina NovaSeq platform (Illumina, USA). Tieguanyin was used as the reference genome [20] using HISAT2 software. Differential gene expression analysis was performed using DESeq2, with multiple hypothesis testing corrected using the Benjamini-Hochberg method to adjust *P*-values and FDR. Differentially expressed genes were filtered based on |log_2_(Fold Change)| ≥ 1 and *P-*adj ≤ 0.05. ClusterProfiler software was used to perform KEGG pathway enrichment analysis on differentially expressed gene sets, while WGCNA (Weighted Gene Co-expression Network Analysis) was conducted using the WGCNA R package. The raw RNA-Seq data generated in this study have been deposited in the National Center for Biotechnology Information (NCBI) Sequence Read Archive (SRA) database under the BioProject accession number PRJNA1450797.

### qRT-PCR validation

Primers for qRT-PCR were designed using Primer Premier 5 software (sequences are presented in Table S1). RNA reverse transcription was performed using PrimeScript™ RT Master Mix (Takara, Japan). Using cDNA from different tea plants as templates, qRT-PCR was performed using the ChamQ Universal SYBR qPCR Master Mix (Vazyme, China) on a Bio-Rad CFX Connect system (Bio-Rad, USA). The reaction conditions were as follows: 95 °C for 30 s; 95 °C for 10 s, and 60 °C for 30 s, repeated 40 times with three biological replicates. Relative expression levels were calculated using the 2^−ΔΔCT^ method. *Cs18s* served as the reference gene, and LJ43 served as the reference sample group.

### Statistical analysis and data visualization

PCA, OPLS-DA, cluster analysis, differential accumulation metabolite screening, correlation analysis, and heatmap generation were performed using Metware Cloud Tools (https://cloud.metware.cn). KEGG enrichment analysis of differentially accumulated metabolites was conducted using MetaboAnalyst 6.0 (www.metaboanalyst.ca). Line graphs, bar charts and statistical analyses (including Student’s t-test and Two-way ANOVA with multiple comparison tests) were generated using GraphPad Prism version 10. Correlation networks were visualized using Cytoscape software. For the evolutionary and structural analysis of *UGTs*, phylogenetic trees were constructed using MEGA 11. Conserved motifs were identified using the MEME suite website (https://meme-suite.org/meme/). The integrated visualization of the phylogenetic tree and conserved domain structures was generated using TBtools-II. The metabolic biosynthesis pathway diagrams were created using Adobe Illustrator 2025.

## Results

### The unique morphological characteristics of RCBMC

**Fig. 1:**
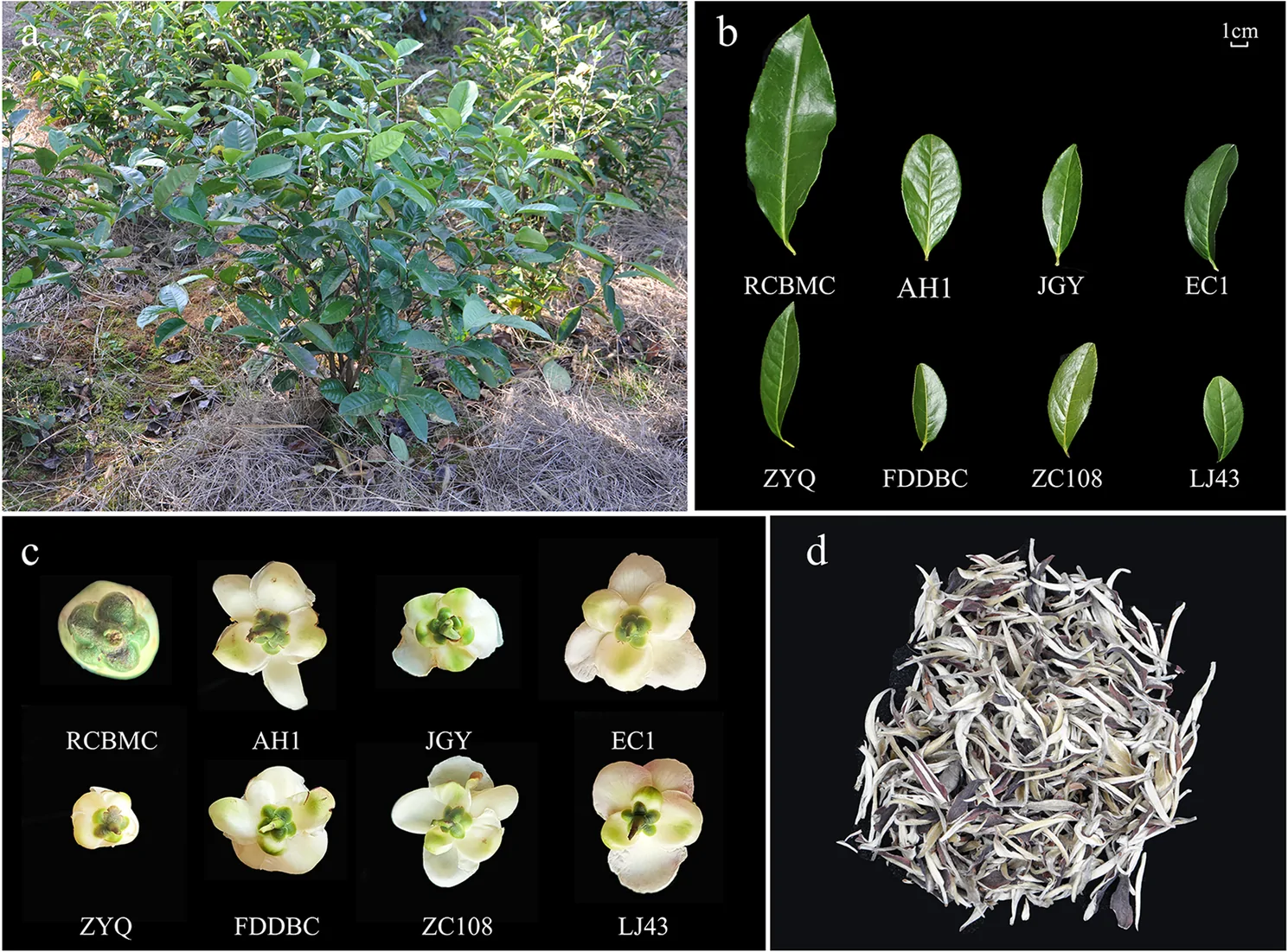
Phenotypic differences between RCBMC and seven cultivars. **a** RCBMC plant. **b** Mature leaves from eight samples. **c** Calyxes from eight samples. **d** White tea processed from RCBMC

Morphological observations indicated significant differences between RCBMC and the seven cultivars. The plant morphology of RCBMC is shown in Fig. 1a. RCBMC produced larger leaves with a stiffer texture and thicker wax layer on the leaf surface (Fig. 1b). Additionally, the calyx surface of RCBMC demonstrated pubescence, whereas all cultivated varieties tested exhibited smooth, hairless calyces (Fig. 1c). The most distinctive feature was the abundant silvery-white trichomes on the underside of its young buds and leaves, which are particularly prominent in white tea processed from RCBMC (Fig. 1d) and may represent physiological adaptations to specific environmental conditions or underlie secondary metabolic processes.

### Metabolic differences between RCBMC and CK cultivars

To comprehensively investigate the differential metabolites between RCBMC (three RCBMC individuals) and CK (JGY, LJ43, ZC108, FDDBC, ZYQ, AH1, and EC1) cultivars, non-targeted metabolomic analysis of tea specimens was conducted using UHPLC-QTOF/MS technology. Following raw data pre-processing, 2,239 compound ions were detected. By comparison with compound databases and integration of retention times from reference standards and secondary mass spectrometry information, 173 compounds were identified in both positive and negative ion modes. These compounds comprised diverse categories, including flavonoids, phenolic acids, alkaloids, and amino acids. The metabolite peaks are shown in Table S2.

**Fig. 2:**
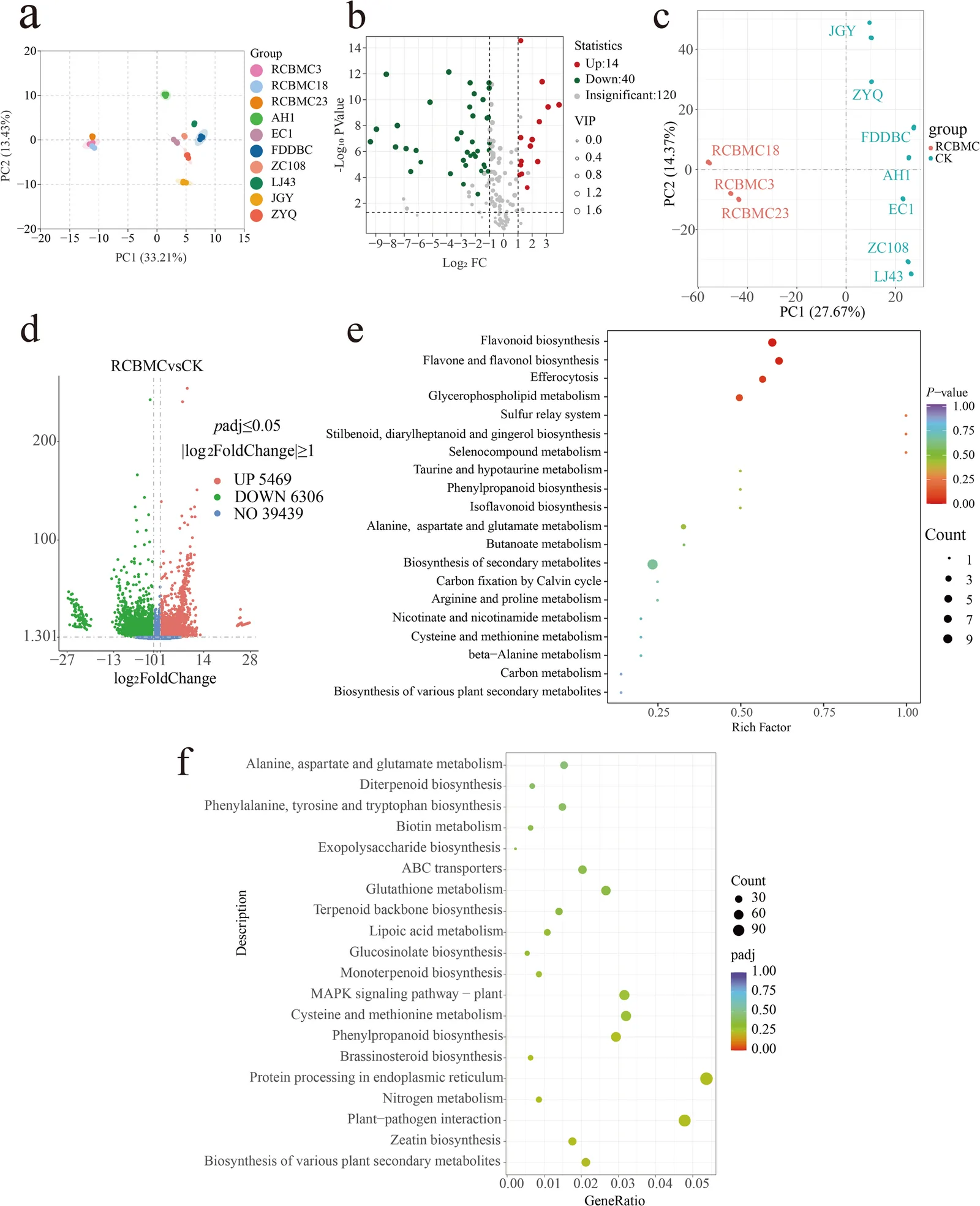
Metabolomics and transcriptome analysis results. **a** PCA analysis results for 10 sample groups. Triangles represent three individual plants of the RCBMC population, and circles represent seven CK cultivars. **b** Volcano plot for DAMs. Red and green dots denote significantly up-regulated and down-regulated metabolites, while grey dots represent non-significant features. The size of the dots corresponds to the Variable Importance in Projection (VIP) score. **c** PCA results for the transcriptome. **d** Volcano plot of DEGs. Red dots represent significantly up-regulated genes, green dots represent down-regulated genes, and blue dots indicate genes with no significant differential expression based on the thresholds of |log₂FoldChange| ≥ 1 and *P-*adj ≤ 0.05. **e** KEGG enrichment results for DAMs. The size of the bubbles indicates the number of DAMs annotated to a specific pathway, and the color gradient from red to blue represents the decreasing level of significance (*P*-value). **f** KEGG enrichment results for DEGs. The dot size represents the number of genes associated with each pathway, and the color scale reflects the adjusted *P*-value, with warmer colors (red) indicating higher statistical significance

**Fig. 3:**
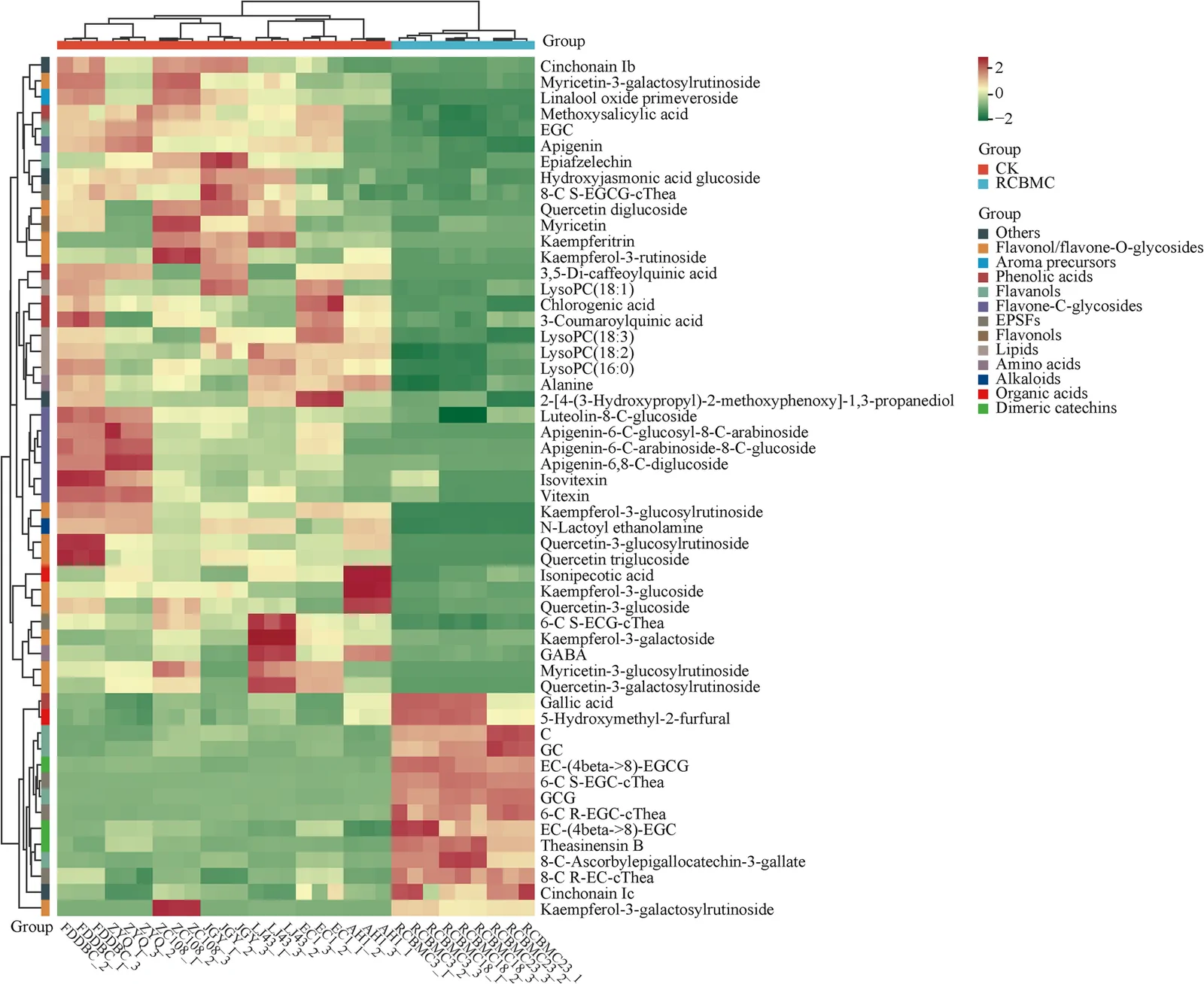
Hierarchical clustering heatmap of differentially accumulated metabolites (DAMs) between RCBMC and CK cultivars. Each column represents an individual sample, and each row represents a specific DAM. The relative abundance of each metabolite is normalized and depicted by a color gradient: red indicates higher accumulation levels, whereas green indicates lower accumulation levels

Multivariate statistical analysis was performed on the relative contents of the identified compounds. PCA revealed that the samples within the groups clustered closely together, indicating high experimental reproducibility. RCBMC and CK showed an apparent separation along the PC1 axis, indicating differences in their overall metabolic profiles (Fig. 2a). The OPLS-DA model further confirmed metabolic differences between the two groups (Fig. S1a). The OPLS-DA permutation test results (Fig. S1b) indicated that the model was stable and reliable, with no signs of overfitting. To further analyze the differences in specific chemical components between RCBMC and CK, 54 differentially accumulated metabolites (DAMs) were screened using thresholds of fold change (FC) > 2 or < 0.5, *P* < 0.05, and variable importance in projection (VIP) > 1. Specifically, 14 metabolites were upregulated in RCBMC, while 40 were downregulated (Fig. 2b). The clustering heatmap visually illustrates the accumulation patterns of differential metabolites across the 10 samples. Specifically, non-epicatechins such as C, CG, and GCG, along with compounds such as gallic acid and 5-Hydroxymethyl-2-furfural, exhibited increased concentrations in RCBMC (Fig. 3). Conversely, most flavonoid glycosides, EGC, and alanine accumulated more abundantly in the CK cultivars. KEGG pathway enrichment analysis revealed that differentially accumulated metabolites were significantly enriched in flavone, flavonol, and flavonoid biosynthesis pathways (Fig. 2e). This indicated that flavonoids represented the core metabolic differences between RCBMC and CK cultivars.

Quantitative analysis of the catechin components in the 10 samples was conducted using HPLC, and the results are presented in Table 1. Consistent with the metabolomic findings, non-epicatechins were higher in RCBMC than in the CK cultivars.

**Table 1:** Catechin components content in 10 samples

Sample	GC(mg·g^− 1^)	EGC(mg·g^− 1^)	C(mg·g^− 1^)	EC(mg·g^− 1^)	EGCG(mg·g^− 1^)	ECG(mg·g^− 1^)	CG(mg·g^− 1^)	GCG(mg·g^− 1^)
RCBMC3	0.60 ± 0.01^c^	8.10 ± 0.31^e^	4.61 ± 0.15^b^	3.02 ± 0.17^de^	120.88 ± 2.34^ab^	26.95 ± 0.33^cde^	2.90 ± 0.10^c^	35.98 ± 0.28^b^
RCBMC18	0.74 ± 0.01^b^	5.88 ± 0.30^e^	4.96 ± 0.55^b^	2.93 ± 0.10^e^	115.59 ± 1.45^bc^	22.89 ± 0.12^de^	6.26 ± 0.15^a^	41.32 ± 0.18^a^
RCBMC23	1.17 ± 0.02^a^	6.76 ± 0.23^e^	5.91 ± 0.06^a^	3.25 ± 0.15^cde^	69.55 ± 1.82^d^	18.90 ± 0.54^e^	5.09 ± 0.43^b^	34.73 ± 0.97^c^
AH1	0.18 ± 0.01^fg^	15.08 ± 0.92^d^	1.54 ± 0.10^c^	4.81 ± 0.84^bcde^	138.38 ± 3.57^a^	28.43 ± 0.48^bcd^	0.62 ± 0.60^d^	1.30 ± 0.11^d^
EC1	0.55 ± 0.01^d^	24.20 ± 1.08^ab^	1.55 ± 0.09^c^	5.70 ± 1.14^bc^	120.62 ± 3.78^ab^	24.77 ± 0.95^cde^	0.37 ± 0.41^d^	1.09 ± 0.10^d^
FDDBC	0.14 ± 0.01^g^	19.52 ± 0.89^c^	1.33 ± 0.25^c^	8.51 ± 0.82^a^	114.31 ± 3.92^bc^	33.09 ± 1.21^bc^	0.72 ± 0.19^d^	1.00 ± 0.07^d^
LJ43	0.25 ± 0.01^e^	18.50 ± 0.61^cd^	1.84 ± 0.23^c^	6.24 ± 0.77^ab^	95.93 ± 2.84^c^	41.64 ± 0.56^a^	0.44 ± 0.19^d^	0.73 ± 0.10^d^
ZC108	0.27 ± 0.04^e^	22.25 ± 3.00^bc^	1.58 ± 0.21^c^	5.50 ± 1.44^bcd^	104.24 ± 17.52^bc^	32.19 ± 5.29^bcd^	0.52 ± 0.11^d^	1.25 ± 0.51^d^
JGY	0.23 ± 0.02^ef^	18.49 ± 2.01^cd^	1.70 ± 0.12^c^	5.58 ± 1.27^bc^	120.67 ± 14.64^ab^	34.43 ± 4.58^b^	0.60 ± 0.79^d^	0.98 ± 0.28^d^
ZYQ	0.22 ± 0.01^ef^	27.13 ± 1.4^a^	1.75 ± 0.39^c^	7.09 ± 0.65^ab^	125.28 ± 3.89^ab^	24.73 ± 1.42^cde^	0.29 ± 0.27^d^	1.44 ± 0.32^d^

Data are presented as the mean ± standard deviation (SD) of three independent biological replicates (*n* = 3). Different lowercase letters within the same column indicate significant differences among the samples at *P* < 0.05, according to one-way ANOVA followed by Duncan’s multiple range test

### Transcriptomic characteristics of RCBMC and CK cultivars

To systematically identify differentially expressed genes (DEGs) between RCBMC and CK cultivars, transcriptome sequencing libraries were constructed with three biological replicates. Thirty samples were sequenced using the Illumina NovaSeq platform. After quality control filtering, each library produced approximately 5.81–8.08 Gb of clean reads, with Q20 and Q30 values exceeding 99% and 96%, respectively. GC content ranged from 39.66% to 44.63%, indicating reliable data quality suitable for subsequent analysis (Table S3).

The heat map of gene expression correlations among the samples indicated high intragroup repeatability (Fig. S2). PCA further revealed that PC1 and PC2 explained 27.67% and 14.37% of the total variance, respectively. Samples from RCBMC and CK cultivars showed a distinct separation along the PC1 axis (Fig. 2c), indicating significant transcriptional differences between them.

Using |log₂(Fold Change)| ≥ 1 and *P-*adj ≤ 0.05 as thresholds, 11,775 DEGs were identified (Table S4). Specifically, 5,469 were upregulated in RCBMC, while 6,306 were downregulated (Fig. 2d). The KEGG pathway enrichment analysis revealed that these DEGs were most significantly enriched in the “Biosynthesis of various plant secondary metabolites” pathway (Fig. 2f). This finding corroborates the metabolomic results, providing transcriptional-level evidence for the extensive chemical composition differences between RCBMC and CK cultivars.

Additionally, DEGs were significantly enriched in the “zeatin biosynthesis” pathway. Further analysis revealed that multiple genes involved in the terpenoid skeleton and adenine synthesis within this pathway were downregulated in RCBMC (Fig. S3), indicating potentially lower endogenous cytokinin levels than those of commonly cultivated varieties. This provides a possible molecular explanation for the slow growth phenotype observed in field studies of RCBMC. DEGs were also significantly enriched in pathways such as “plant pathogen interaction” and “nitrogen metabolism,” indicating that RCBMC and CK cultivars may exhibit differences in regulatory mechanisms regarding responses to biotic stress and nitrogen use efficiency.

### Analysis of gene expression patterns in the flavone and flavonol biosynthesis pathway

Metabolomic data revealed that differential metabolites were significantly enriched in the flavone and flavonol biosynthesis pathway. Targeted quantitative analysis indicated differences in the catechin components between RCBMC and CK cultivars. KEGG enrichment analysis of DEGs revealed that the flavone and flavonol biosynthesis pathway was not significantly enriched. In plant secondary metabolite networks, significant changes in metabolite accumulation often do not require global transcriptional activation of the entire pathway. Changes in the transcriptional levels of key enzyme genes within the metabolic pathway are sufficient to alter metabolic flux, thereby leading to differential metabolite accumulation [21, 22].

**Fig. 4:**
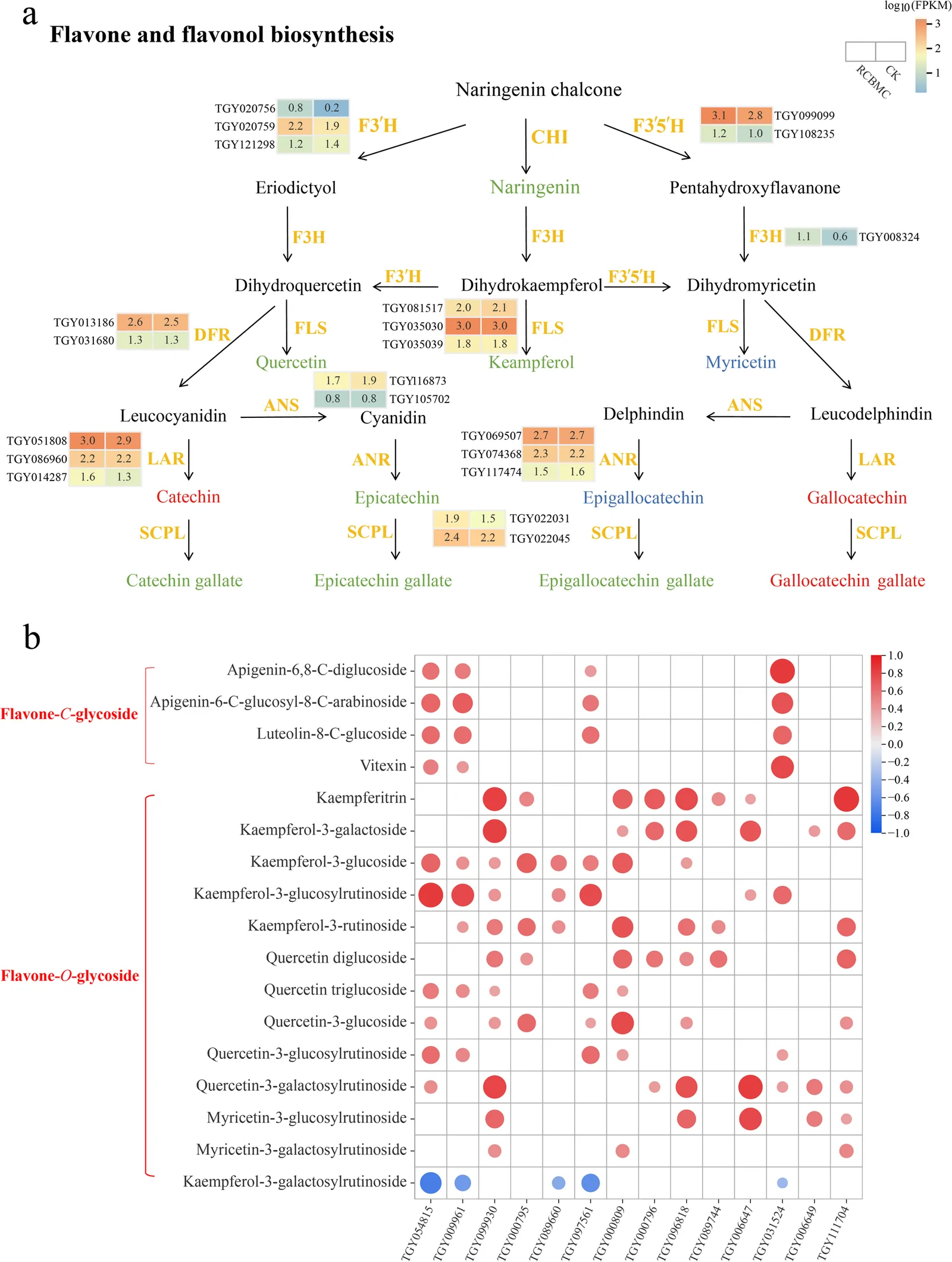
Expression profiles of structural genes and *UGTs* regulating differential flavonoid accumulation in RCBMC. **a** Heatmap of flavone and flavonol biosynthesis pathways. The grids adjacent to the enzymes show the gene expression heatmaps comparing RCBMC with the CK cultivars. Black indicates metabolites belonging to the flavone and flavonol biosynthesis pathway but not detected in the metabolome. Green represents metabolites showing no difference between the two groups. Red represents metabolites upregulated in RCBMC. Blue indicates metabolites downregulated in RCBMC. Orange highlights key synthase genes within the flavone and flavonol biosynthesis pathway. CHI, chalcone isomerase; F3′H, flavonoid 3′-hydroxylase; F3′5′H: flavonoid 3′5′-hydroxylase; FNS, flavone synthase; F3H, flavonoid 3-hydroxylase; FLS, flavonol synthase; DFR, dihydroflavonol 4-reductase; ANS, anthocyanidin synthase; ANR, anthocyanidin reductase; LAR, leucoanthocyanidin reductase; SCPL, serine carboxypeptidase-like acyltransferase. **b** Correlation map of differentially expressed *UGTs* and differentially accumulated flavonoid glycosides. The color of the circles within the grid represents the level of correlation: red indicates a positive correlation, while blue indicates a negative correlation. Larger circles and darker colors represent higher absolute correlation values. The figure only shows results with *P* < 0.05

This study further analyzed the transcript levels of key structural genes in this pathway (Fig. 4a), including *F3’H*, *F3’5’H*, *F3H*, *FLS*, *DFR*, *LAR*, *ANS*, and *ANR*. The upstream genes *F3’5’H* and *F3H* showed mild upregulation in RCBMC, potentially providing richer precursors for downstream metabolite synthesis. Midstream genes such as *DFR*, *FLS*, and *ANR* exhibited similar overall transcript levels between the two groups. Notably, gene expression in the downstream competitive pathway was significantly different. The *ANS* gene (TGY116873), which catalyzes the conversion of colorless anthocyanins into anthocyanins, was downregulated in RCBMC. Conversely, the *LAR* gene (TGY051808 and TGY014287), which catalyzes the conversion of leucoanthocyanidins to non-epicatechins, was upregulated. This indicates that the metabolic flux in RCBMC is redirected toward non-epicatechins dominated by *LAR* while reducing the biosynthesis of *ANS*-dominated epimeric catechins. Consequently, non-epicatechins accumulated at higher levels in RCBMC than in the CK. These findings also indicate that the specific accumulation of flavonoid metabolites in RCBMC is not driven by overall transcriptional activation or repression of the pathway, but rather by the imbalanced expression of key structural genes within the pathway.

Similar to the accumulation patterns of C, GC, and GCG, we found that the content of CG in RCBMC was significantly increased by approximately 20-fold compared to the CK cultivars (Table 1). We further analyzed the transcript levels of galloyltransferases gene. In tea plants, the galloylation of catechins is primarily catalyzed by *CsSCPL4* (TGY022031) and *CsSCPL5* (TGY022045) [23]. Transcriptomic analysis revealed that *CsSCPL4* was significantly upregulated in RCBMC. Meanwhile, although *CsSCPL5* was not identified as a differentially expressed gene based on the FC > 2 threshold, its FPKM in RCBMC was still slightly higher than that in CK cultivars. This indicates that RCBMC possesses a higher galloylation capacity.

Therefore, the significant accumulation of CG and GCG in RCBMC is associated with the high accumulation of upstream precursors. In RCBMC, the upregulation of *LAR* and downregulation of *ANS* significantly redirected metabolic flux toward non-epicatechins while limiting the production of epi-catechins. Downstream galloyltransferases *CsSCPLs* primarily use C and GC as substrates to convert them into CG and GCG. The reduction in epi-catechin precursors resulted in EGCG not showing a corresponding proportional increase in RCBMC.

### Investigating glycosyltransferase expression identifies downregulated *UGTs* associated with low flavonoid glycosides in RCBMC

Metabolomic analysis revealed that most flavonoid glycosides in RCBMC (except kaempferol-3-galactosylrutinoside) were significantly downregulated. To elucidate this phenomenon at the transcriptional level, we focused on the final critical step in flavonoid glycoside biosynthesis (glycosylation modification) and conducted further analysis of the glycosyltransferase genes catalyzing this reaction. Seventeen uridine diphosphate glycosyltransferases **(***UGTs*) were significantly downregulated in RCBMC.

We performed a conserved domain analysis on the 17 downregulated candidate *UGT*s initially screened and excluded three genes lacking a complete UGT domain. The remaining 14 candidate genes all contained typical Glycosyltransferase-GTB-type or MGT superfamily domains (Fig. S4). Multiple sequence alignment results showed that all 14 candidate genes contained a highly conserved PSPG box, structurally confirming their classification as *UGTs* (Fig. S5). To further predict their functions, we selected 20 known *O*-glycosyltransferases and 14 known *C*-glycosyltransferases (Gene accession numbers are listed in Table S5)to construct a phylogenetic tree and perform MEME motif analysis alongside the candidate genes. The phylogenetic and motif analyses corrected our previous inferences. Among the 14 candidate genes, only one gene (TGY009961) clustered closely with known *C*-glycosyltransferases and shared the same motifs with them. The remaining 13 candidate *UGTs* were all clustered within the *O*-glycosyltransferase branch (Fig. S6).

To confirm their direct association with flavonoid glycoside accumulation, we correlated their transcript levels with those of the differentially expressed flavonoid glycosides. The results showed that the expression levels of all 14 *UGTs* were significantly and positively correlated with flavonoid glycoside content, except for kaempferol-3-galactosylrutinoside. This is likely the key reason for the low flavonoid glycoside content in RCBMC (Fig. 4b). Although phylogenetic analysis classified only TGY009961 as a *C*-glycosyltransferase, the other three candidate *UGTs* still showed a strong positive correlation with the accumulation of flavonoid-*C*-glycosides. Since transcriptomic correlations typically reflect transcriptional synchrony rather than direct enzymatic relationships, and studies have shown that the boundary between *O*- and *C*-glycosylation in plant *UGTs* is not strictly absolute. Several characterized UGTs (e.g., MiCGT and FcCGT) inherently possess, or exhibit through fine-tuned regulation, non-specific bifunctionality, enabling them to efficiently catalyze both *O*- and *C*-glycosylation reactions depending on substrate binding patterns [24]. Therefore, the potential for *C*-glycosylation activity in vivo by these three *UGTs* cannot be ruled out.

### WGCNA analysis

To systematically elucidate the transcriptional regulatory network influencing flavonoid metabolism, this study employed weighted gene co-expression network (WGCNA) analysis based on transcriptome data. First, low-expression genes were filtered across all samples using an FPKM > 0 threshold, resulting in 25,878 genes for network construction. Soft thresholding validation indicated that a power of 12 achieved a fit index > 0.8, meeting the scale-free network criterion and ensuring the reliability of the network (Fig. S7a). We constructed a network based on the determined soft thresholds. Hierarchical clustering was performed using the dissimilarities between genes to establish a hierarchical clustering tree. Dynamic pruning was applied to segment the tree into distinct modules (minimum module size: 30 genes). Modules with correlation coefficients > 0.75 (dissimilarity coefficients < 0.25) were merged to produce 39 gene modules (Fig. S7b).

**Fig. 5:**
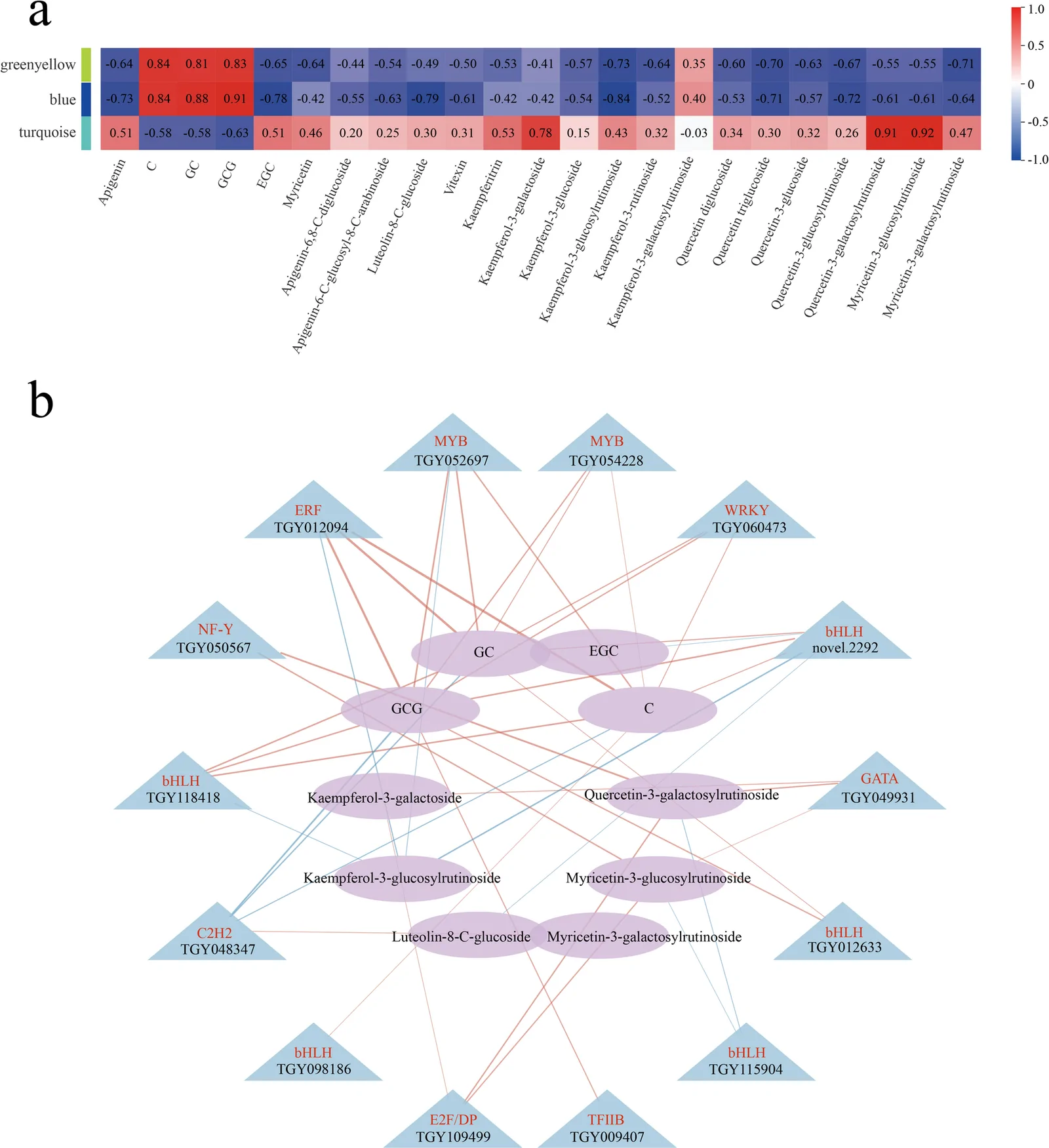
WGCNA module-trait relationships and the transcription factor-metabolite regulatory network. **a** Module-trait relationship. The x-axis represents flavonoid metabolites, while the y-axis represents gene modules. Each cell contains the corresponding correlation coefficient and *P*-value, with the *P*-value indicated in parentheses. **b** Transcription factor-metabolite correlation network. Blue triangles represent transcription factor genes, purple ovals indicate differentially expressed flavonoid metabolites, and connecting lines indicate correlation coefficients between transcription factor expression levels and flavonoid content. Blue lines represent negative correlations, and red lines indicate positive correlations. The line width increases as |*r*| approaches 1 and decreases otherwise

A comprehensive correlation analysis was initially performed between all 39 modules and differentially accumulated flavonoids, with the complete module-trait heatmap provided in the supplementary data (Fig. S8). This analysis revealed that the green-yellow and blue modules showed significant positive correlations with C, GC, and GCG content (correlation coefficient > 0.8 and *P* < 0.05). The turquoise module showed significant positive correlations with quercetin-3-galactosylrutinoside and myricetin-3-glucosylrutinoside content (correlation coefficient > 0.8 and *P* < 0.05). Based on these findings, these three modules were selected for subsequent analyses (Fig. 5a).

From each module, the top 10% genes based on the kWithin value were selected as core genes, resulting in 693 candidate genes, including 18 transcription factors.

To elucidate the regulatory relationships between these transcription factors and flavonoid metabolites, we constructed a correlation network linking gene expression levels and metabolite contents, retaining only significant associations with |*r*| > 0.8 and *P* < 0.05. The final network comprised 14 transcription factors and multiple differentially expressed metabolites. These transcription factors covered multiple families, including: WRKY (TGY060473), NF-Y (TGY050567), MYB (TGY052697, TGY054228), GATA (TGY049931), ERF (TGY012094), E2F/DP (TGY109499), C2H2 (TGY048347), bHLH (novel.2292, TGY118418, TGY012633, TGY098186, TGY115904), and transcription initiation factor TFIIB (TGY009407). This correlation network revealed complex regulatory patterns in which the same transcription factor exerted opposing regulatory effects on different metabolites. For example, the *ERF* transcription factor TGY012094 showed a significant positive correlation with catechin C, GC, and GCG content, but a negative correlation with kaempferol-3-glucosylrutinoside (Fig. 5b).

All 14 core transcription factors highlighted by the co-expression network were significantly differentially expressed between RCBMC and the CK cultivars (*P* < 0.05), and their expression changes were consistent with the associated metabolite accumulation patterns (Fig. S9).

### Variations in strictinin content across different tea plant germplasms and seasons

We also identified strictinin in the metabolomics data. In the OPLS-DA model, the VIP score of strictinin was < 1 (0.637), largely because multivariate statistical models in tea metabolomics are typically dominated by the variances of highly abundant secondary metabolites (e.g., major catechins and alkaloids). However, univariate analysis revealed a significant accumulation of strictinin in RCBMC compared with the CK cultivars, exhibiting a fold-change of 2.27 (log2FC > 1). Although strictinin did not meet the conventional multivariate VIP threshold for differentially expressed metabolites, it was selected as a core research target due to its significant physiological value. Because strictinin was present in all tested cultivars but distinctly enriched in RCBMC, we sought to determine whether this high-accumulation trait is widely distributed among tea germplasms or strictly dependent on specific genetic backgrounds.

**Fig. 6:**
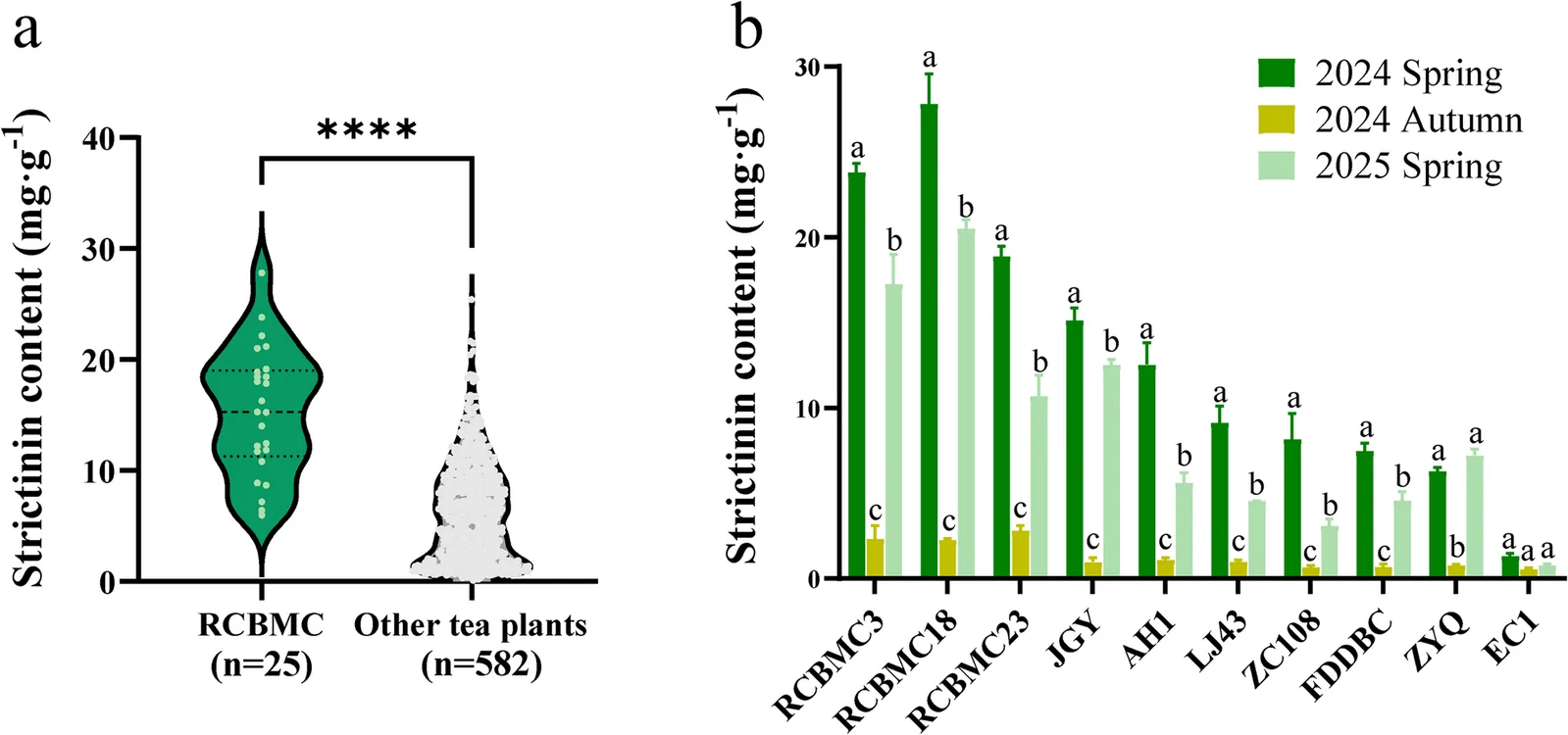
Extraordinary accumulation and seasonal variation of strictinin in the RCBMC population. **a** Violin plot comparing the strictinin content between the RCBMC population (*n* = 25) and a large-scale panel of other tea germplasms (*n* = 582). The white dots represent individual samples. The dashed lines within the violins indicate the median and upper/lower quartiles. Asterisks indicate a highly significant difference between the two groups based on Student’s t-test (**** *P* < 0.0001). **b** Seasonal and interannual dynamic changes in strictinin content among three representative RCBMC individuals and seven CK cultivars. Green, yellow, and light green bars represent samples harvested in Spring 2024, Autumn 2024, and Spring 2025, respectively. Data are expressed as the mean ± standard deviation (SD) of three biological replicates. Different lowercase letters above the bars indicate statistically significant differences across different seasons within the same individual (*P* < 0.05, based on a Two-way ANOVA followed by multiple comparison tests). All contents were calculated based on dry weight (DW)

Therefore, in the spring of 2024, quantitative detection of strictinin was conducted in 607 medium- and small-leaf tea plants, including 25 individual plants from the RCBMC population. Strictinin was present in all samples, with the RCBMC population exhibiting significantly higher levels of strictinin than the other medium- and small-leaf tea plants (Fig. 6a). As presented in Table 2, strictinin levels in the RCBMC population ranged from 6.18 to 29.71 mg·g⁻¹, with an average of 15.35 mg·g⁻¹. In other medium- and small-leaf tea plants, strictinin levels ranged from 0.22 to 19.70 mg·g⁻¹, with an average of 8.23 mg·g⁻¹.

**Table 2:** Content of strictinin in tea plants

Component	Sample	Mean ± SD	Maximum (mg·g^− 1)^	Minimum (mg·g^− 1)^	CV (%)
		(mg·g^− 1)^			
Strictinin	RCBMC	15.35 ± 5.65****	29.71	6.18	42.98
	Other tea plants	8.23 ± 3.34	19.70	0.22	40.53

“RCBMC” represents individuals from the ‘Rucheng Baimaocha’ population (*n* = 25), while “Other tea plants” represents the remaining diverse tea germplasms (*n* = 582). Data are expressed based on dry weight (DW)

*Abbreviations:* *SD *standard deviation, *CV *coefficient of variation

****indicates a highly significant difference between the two groups at P < 0.0001 (Student’s t-test)

Additionally, quantitative analysis of strictinin was conducted in the autumn of 2024 and spring of 2025 on three individual plants of RCBMC and seven cultivars used for this transcriptomic and metabolomic analysis, and the results are shown in Fig. 6b. In spring 2025, the strictinin content in different samples was slightly lower than that in spring 2024; however, the overall trend of varying content across samples remained consistent. To evaluate the effects of genetics and season, a two-way analysis of variance was performed. The analysis quantified that genetic background and seasonal factors accounted for 40.68% and 41.01% of the variation in strictinin content, respectively, with their interaction accounting for 17.68% (*P* < 0.0001). This indicates that strictinin accumulation is a trait co-regulated to nearly equal degrees by both genetic background and season. Multiple comparison tests confirmed that the strictinin content in autumn was significantly lower than that in spring (*P* < 0.05, indicated by different lowercase letters in Fig. 6b), suggesting that its synthesis is strongly responsive to seasonal environmental shifts.

### Identification of candidate genes for the biosynthesis of strictinin

Strictinin is a structurally simple ellagitannin formed by adding galloyl groups to positions 1, 4, and 6 of uridine diphosphate (UDP)-glucose, with galloyl groups at the 4- and 6-positions by oxidatively coupling to create the final structure. Based on its structural formula, strictinin biosynthesis is hypothesized to involve four distinct reactions. These include three reactions common to the synthesis of hydrolyzable tannins and one reaction unique to the formation of ellagitannins.

**Fig. 7:**
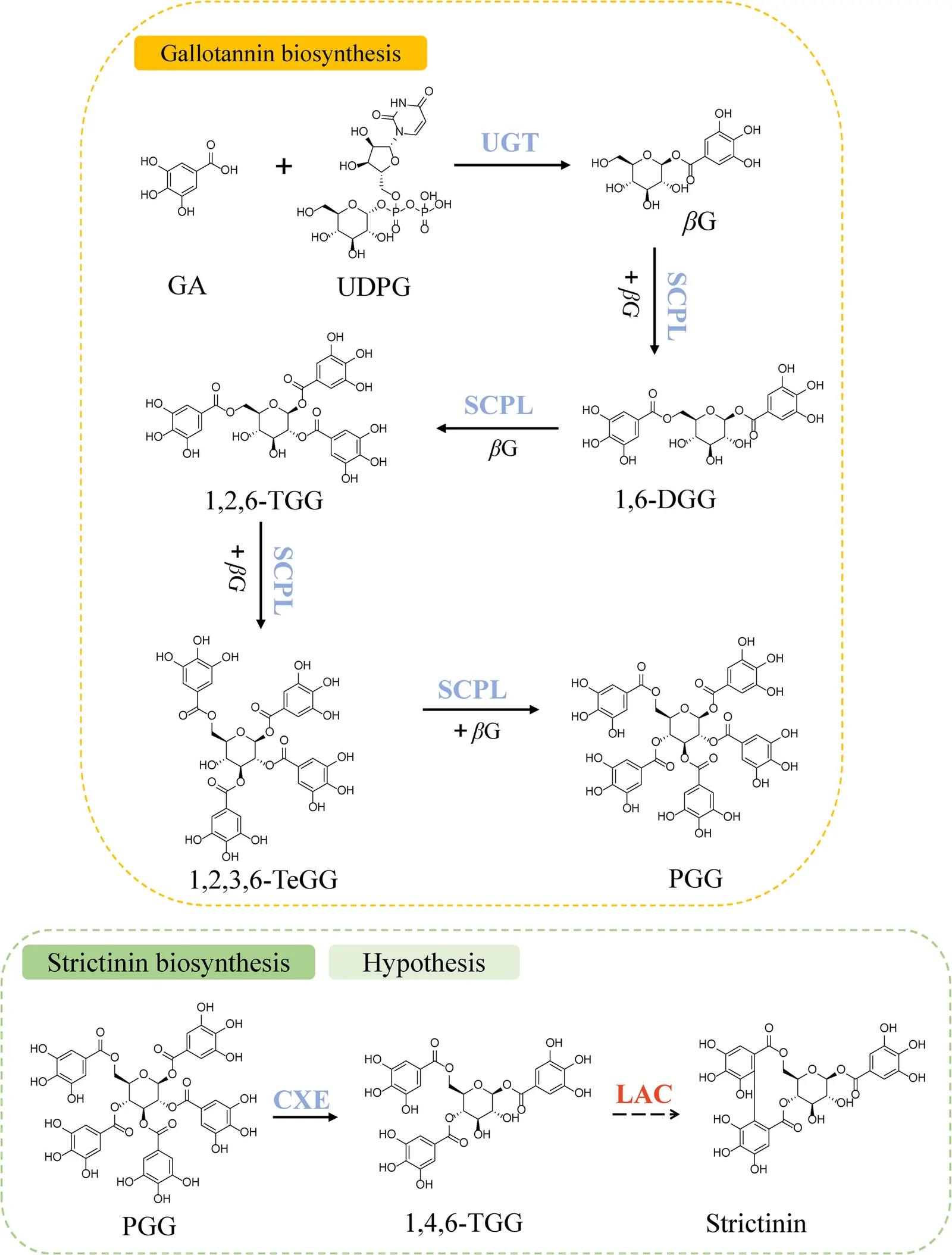
Prediction of the biosynthesis pathway of strictinin. UGT, uridine diphosphate glycosyltransferases; SCPL, serine carboxypeptidase-like; CXE, carboxylesterases; LAC, laccase. GA, gallic acid; UDPG, uridine diphosphate glucose; *β*G, *β*-D-glucosidase; 1,6-DGG, 1,6-Di-*O*-galloyl-*β*-D-glucose; 1,2,6-TGG, 1,2,6-Tri-*O*-galloyl-*β*-D-glucose; 1,2,3,6-TeGG, 1,2,3,6-Tetra-*O*-galloyl-*β*-D-glucose; PGG, 1,2,3,4,6-Pentakis-*O*-galloyl-*β*-D-glucose; 1,4,6-TGG, 1,4,6-Tri-*O*-galloyl-*β*-D-glucose

The predicted biosynthetic pathway of strictinin is shown in Fig. 7. Gallic acid (GA) and uridine diphosphate glucose (UDPG) are precursors of strictin biosynthesis. Under the catalysis of UGT, they form 1-*O*-galloylglucose (*β*G). Subsequently, *β*G undergoes four consecutive galloylation reactions catalyzed by serine carboxypeptidase-like (SCPL) to produce pentagalloylglucose (PGG). The galloyl group in PGG is hydrolyzed by carboxylesterase (CXE), resulting in 1,4,6-tri-*O*-galloylglucose (1,4,6-TGG). 1,4,6-TGG is oxidatively coupled with laccase (LAC) to form strictinin.

**Fig. 8:**
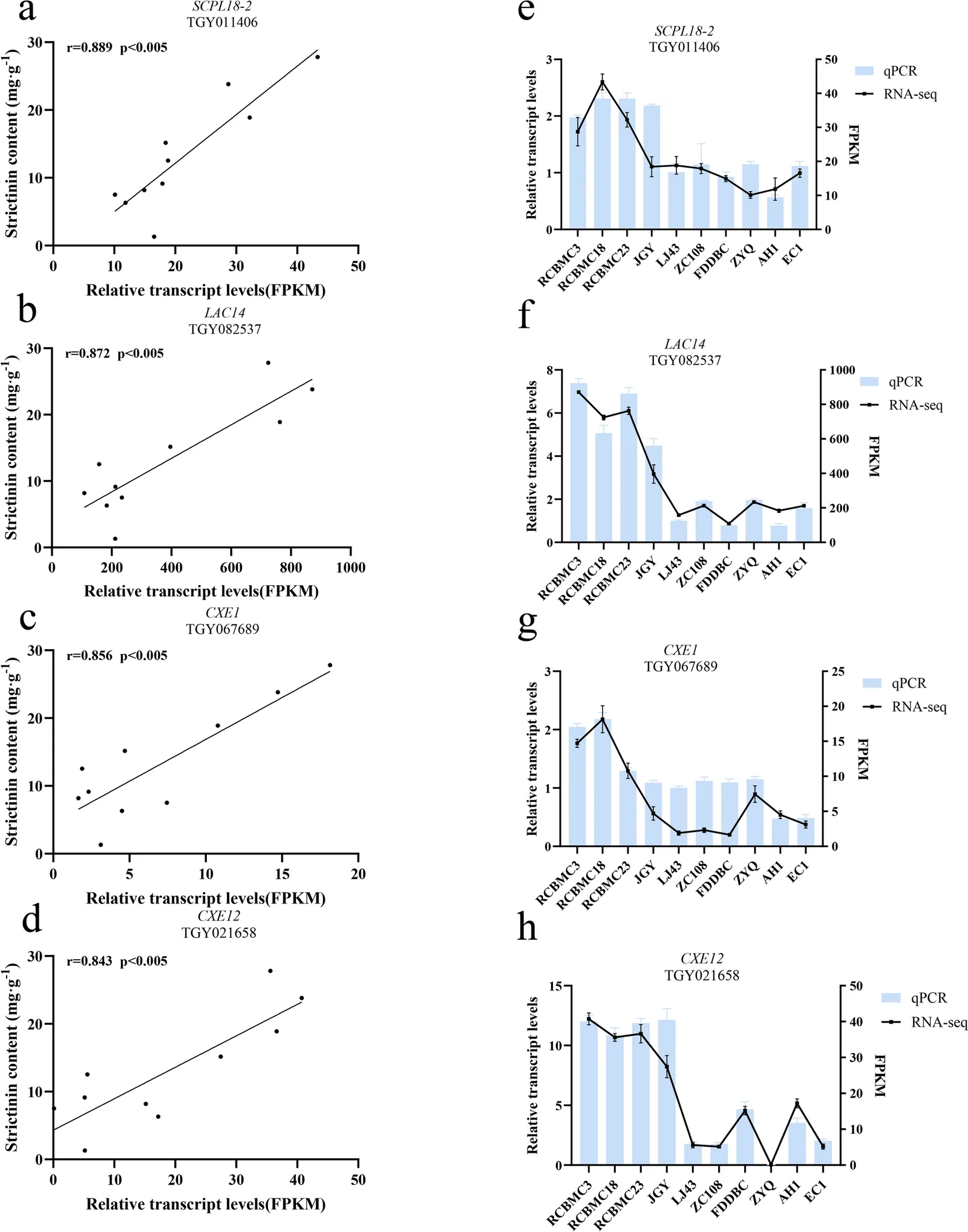
Screening of genes related to strictinin biosynthesis. **a**–**d** Correlation between gene relative transcript levels (FPKM) and strictinin content. **e**–**h** Candidate gene qPCR validation results

Among the DEGs, *SCPL*, *CXE*, and *LAC* were identified. Correlation analysis was performed between gene FPKM values and strictinin content in the spring of 2024. Four genes with correlation coefficients *r* > 0.8 for strictinin content were identified: one *SCPL* (TGY011406), one *LAC* (TGY082537), and two *CXEs* (TGY067689 and TGY021658). These genes were validated by qPCR. qPCR validation results for these four genes were largely consistent with the transcriptome data, indicating their potential involvement in strictinin biosynthesis (Fig. 8). To validate the reliability of the transcriptome data, four additional *SCPL*, *LAC*, and *CXE* genes were screened using qPCR. These results were consistent with the transcriptome data, indicating the reliability of the transcriptome data (Fig. S10).

## Discussion

### The metabolic characteristics of fresh leaves in RCBMC

Metabolomic analysis indicated that the core difference between RCBMC and CK cultivars is in their unique flavonoid metabolic profiles. Flavonoids are a major class of secondary metabolites that typically exist as glycosides [25]. Flavonoid glycosides impart bitter taste and astringency to tea [26]. The flavonoid glycoside content in RCBMC was lower than that in the CK cultivar, indicating that RCBMC is an excellent material for breeding tea plant varieties with reduced bitterness and astringency. Regarding catechin components, RCBMC accumulated increased levels of non-epicatechins, such as C, CG, and GCG. The distribution of catechins and their derivatives varies among *Camellia* species, which is a key basis for taxonomic classification [27, 28]. The composition of catechins is crucial for determining astringency, bitterness, and subsequent flavor development [29]. GCG exhibits lower bitterness and superior antioxidant activity and cholesterol absorption inhibition compared to that of EGCG [30, 31]. This indicates that a high GCG content can enhance the flavor of tea and enhance its health benefits.

Additionally, we observed that RCBMC exhibited high levels of strictinin, an ellagitannin. This has also been detected in plants such as *Casuarina equisetifolia* and *Fragaria × ananassa*; however, current studies on its pharmacological activity and developmental applications have primarily focused on tea plants [32]. Strictinin has beneficial physiological functions, therefore, RCBMC represents a valuable germplasm for cultivating tea varieties rich characterized by naturally high-functional components.

Although this study revealed the metabolic characteristics of RCBMC, certain limitations remain. Metabolite detection in this study relied on database matching, which indicates that unannotated metabolites could not be identified, potentially leading to the omission of certain specific metabolites. Currently, techniques such as infrared (IR) spectroscopy and nuclear magnetic resonance (NMR) spectroscopy have been applied to identify specific components of tea plant germplasm resources [33]. In subsequent studies, these technologies could be utilized to analyze unknown metabolites in RCBMC further, thereby enriching their secondary metabolite profiles.

### Transcriptomics study on metabolic regulation in fresh leaves of RCBMC

Transcriptomic results indicated that DEGs in RCBMC and CK cultivars were significantly enriched in the biosynthesis of various plant secondary metabolite pathways, consistent with the metabolomic findings. However, DEGs were not significantly enriched in the flavone and flavonol biosynthesis pathway. This suggests that metabolite accumulation is not solely influenced by the transcriptional levels of key synthase genes but may also be subject to multi-level regulation at the post-transcriptional and translational levels. However, we identified the reason for the specific accumulation of non-epicatechins in RCBMC as the expression levels of key synthase genes in this pathway: increased *LAR* expression and decreased *ANS* expression together drive metabolic flux toward the synthesis of non-epicatechins. Simultaneously, we identified a series of *UGTs* that were significantly downregulated in RCBMC and positively correlated with flavonoid glycoside accumulation. The identification of these genes elucidates why RCBMC has a low flavonoid glycoside phenotype, as well as provides potential genetic markers for breeding tea plant cultivars with reduced bitterness (low flavonoid glycosides). The core transcription factors identified using WGCNA showed a significant correlation with key metabolite accumulation, providing targets for the future improvement of multiple metabolic traits through the regulation of these key transcription factors.

### Strictinin biosynthetic pathway hypothesis

Although our study indicated the widespread presence of strictinin in tea plants, the biosynthetic pathway of strictinin in tea plants has not yet been fully elucidated, and the key genes involved in its biosynthesis remain unclear. Among the reported biosynthetic pathways for tannins in plants, the *SCPL*, *CXE*, and *LAC* gene families play important roles. SCPL, as a typical acyltransferase, catalyzes the galloylation reaction; CXE is primarily responsible for the hydrolysis of gallate esters; and LAC catalyzes the oxidative coupling of phenolic compounds. Based on the functions of these synthase genes and the chemical structure of the compound, we hypothesize that its biosynthesis primarily involves the following four major reactions:

The first step is the glycosylation of gallic acid. UDP-glycosyltransferase (UGT) catalyzes the combination of GA with UDP-glucose to form 1-*O*-galloylglucose (*β*G), a key intermediate acting as both an acyl donor and acceptor [34].The gene *CsUGT84A22*, involved in *β*G synthesis, has been reported in tea plants, and its catalytic activity has been validated in vitro [35].

The second reaction involves the acylation of gallotannin. The serine carboxypeptidase-like (SCPL) gene family may participate in the acylation of gallotannins. SCPL3 in *Camellia oleifera* catalyzes a sequential four-step galloylation reaction from *β*-glucose to 1,2,3,4,6-pentagalloylglucose (PGG) [36].

The third reaction step involves the hydrolysis of the galloyl groups. During PGG biosynthesis, the substitution sequence of galloyl groups is not random but follows a specific pattern [37, 38]. Because the galloyl group in strictinin is located at the 1,4,6-positions of UDP-glucose, which does not align with the de novo synthesis sequence for galloylglucose, it is hypothesized that hydrolytic cleavage of the galloyl group occurs during strictinin biosynthesis. This process involves the removal of two galloyl groups from PGG to produce a strictin backbone structure. Carboxylesterases (CXEs) are involved in tannin hydrolysis. For example, CsTA in tea plants metabolizes gallic tannin, ellagitannin, and esterified catechins [39]. Subsequent studies have revealed that CsTA represents a class of hydrolases exhibiting mixed acyltransferase activity [40].

The final reaction step involves the oxidative coupling of the galloyl groups. Unlike gallotannins, ellagitannins such as strictinin require the oxidation of galloyl groups to form C-C bonds, thereby generating the characteristic hexahydroxydiphenol (HHDP) group. Laccase (LAC) is the coupling agent for the HHDP group formation [41, 42].

This study preliminarily identified candidate enzyme genes such as *SCPL*, *CXE*, and *LAC* that may be involved in strictinin biosynthesis through a correlation analysis between gene transcription levels and strictinin content, laying the foundation for elucidating this novel metabolic pathway. Although the candidate genes have been validated by qPCR, their functions require confirmation by in vitro enzymatic assays and transgenic plant experiments. Future research should utilize strictinin content data from 607 germplasm resources to conduct genome-wide association studies (GWAS). By employing forward genetic approaches, key genes involved in strictin biosynthesis can be identified and cross-validated with existing candidate genes.

### Seasonal dynamics and genetic-environmental co-regulation of strictinin accumulation

This study found that the accumulation of strictinin in tea plants is a dynamic process, with its content exhibiting significant variation across different seasons. A two-way analysis of variance clearly quantified that seasonal environmental changes (41.01%) and genetic background (40.68%) contribute almost equally to the variation in strictinin content. Previous studies have shown that the final metabolic phenotype of tea plants is determined by the complex interaction between the genetic characteristics of the cultivar and external ecological conditions [43].

Existing studies provide in-depth mechanistic insights into how these environmental signals influence the secondary metabolism of tea plants. For example, low temperatures in early spring or at high altitudes, acting as environmental stress signals, can induce cold acclimation responses in tea plants, thereby altering the metabolic profiles of amino acids and carbohydrates [44, 45]. Furthermore, low-temperature stress can induce the expression of specific transcription factors (such as *CsABF2*), which in turn directly activate the transcription of key structural genes downstream of the phenylpropanoid and flavonoid biosynthetic pathways [46]. The metabolic response of tea plants to environmental factors exhibits significant variety-dependence. Studies have shown that in response to temperature changes, conventional green-leaf varieties and temperature-sensitive albino varieties exhibit significant differentiation in the accumulation patterns of flavonoid glycosides, reflecting the highly complex, environment-dependent metabolic adaptation mechanisms of tea plants [47]. In addition to temperature, specific wavelengths of light can also regulate secondary metabolic pathways in tea plants [48]. In recent years, epigenetic modifications have been recognized as one of the key mechanisms by which seasonal climate changes regulate secondary metabolism. Studies have confirmed that seasonal environmental shifts can alter the DNA methylation status of tea plants, leading to widespread upregulation of the expression of numerous flavonoid biosynthesis-related genes, including *CsPAL*, *CsCHI*, and *CsSCPLs* [49].

Although significant progress has been made regarding the environmental response mechanisms of conventional flavonoids and amino acid metabolism in tea plants, our understanding of the seasonal and genetic co-regulatory mechanisms of strictinin remains limited. We hypothesize that fluctuations in strictinin levels are governed by similar regulatory networks. In future studies, elucidating how key environmental factors such as temperature and photoperiod specifically regulate the expression of key genes involved in strictinin biosynthesis, as well as investigating whether seasonal epigenetic modifications drive the seasonal fluctuations of strictinin, will be crucial research directions for fully elucidating the mechanisms underlying the accumulation of this metabolite.

### Growth–defense tradeoffs and breeding implications in RCBMC

This study also identified findings from metabolome and transcriptome data that were consistent with field phenotypic observations. Numerous studies have indicated that, in addition to influencing tea flavor, the role of flavonoid glycosides in tea plant stress resistance is important. The accumulation of flavonoid glycosides enables tea plants to withstand both biotic and abiotic stresses [50–52]. The reduced flavonoid glycoside content indicates that RCBMC may exhibit weaker stress resistance than cultivated varieties, which is consistent with our field observations of poorer cold tolerance.

Transcriptome data indicated the downregulated expression of genes associated with zeatin biosynthesis in RCBMC, consistent with the slow-growing field phenotype. Owing to its thick leaf wax layer and low flavonoid glycoside content, we hypothesize that RCBMC uses a resource allocation strategy characterized by a “tradeoff between growth and defense.” Under biotic and abiotic stress conditions, plants experiencing resource constraints frequently reallocate more resources toward the activation of their defense systems at the expense of normal growth and development [53]. Additionally, the enrichment of DEGs in the “plant-pathogen interaction” and “nitrogen metabolism” pathways further supports the hypothesis that RCBMC has a unique genetic foundation for stress response and nutrient utilization.

These results suggest that in future breeding practices, balancing the introduction of desirable metabolic traits, such as high strictinin content, against potential negative effects, such as reduced growth vigor and decreased resistance, is necessary while selecting RCBMC as a parental line in hybrid combinations. In F_1_ populations, molecular marker-assisted selection or phenotypic screening should be employed to achieve the optimal integration of target traits with agronomic characteristics.

## Conclusions

This study elucidates the unique metabolic profiles and underlying molecular mechanisms of the ‘Rucheng Baimaocha’ (RCBMC). Compared to cultivated varieties, RCBMC accumulates higher levels of non-epicatechins and strictinin, accompanied by lower levels of flavonoid glycosides. The upregulation of *LAR* and the downregulation of *ANS* and multiple *UGTs* drive this differential metabolite accumulation. Correlation analysis identifies specific members of the *SCPL*, *CXE*, and *LAC* families as candidate genes for strictinin biosynthesis. Furthermore, quantitative screening of 607 tea germplasms and multi-seasonal testing of representative resources indicate that strictinin accumulation is highly dependent on genetic background and regulated by seasonal environmental factors.

This research deepens the scientific understanding of the specific biochemical phenotype of RCBMC. The large-scale quantitative screening establishes the genetic stability and utilization value of RCBMC as an elite resource highly enriched in strictinin. Additionally, transcriptomic data analysis provides candidate genes for unraveling the biosynthetic pathways of tannins, such as strictinin, in tea plants. These findings provide a theoretical basis for developing specialty functional tea products with high health value and lay a solid material and genetic foundation for the precise improvement of tea quality components through future marker-assisted breeding.

## Supplementary Information

Supplementary Material 1.

Supplementary Material 2.

## Data Availability

The raw RNA-Seq data generated in this study have been deposited in the National Center for Biotechnology Information (NCBI) Sequence Read Archive (SRA) database under the BioProject accession number PRJNA1450797 (https://www.ncbi.nlm.nih.gov/bioproject/PRJNA1450797). All other data will be made available on request.
