# Supplementary material for: Integrated metabolomic and transcriptomic analysis of Camellia sinensis var. pubilimba ‘Rucheng Baimaocha’ reveals distinct flavonoid and strictinin biosynthesis
Source: BMC Plant Biol. 2026 Jul 15;26:1733. doi: 10.1186/s12870-026-09521-7 (PMC13651260; doi:10.1186/s12870-026-09521-7)
Supplement: Supplementary file 1 [file 12870_2026_9521_MOESM1_ESM.docx]

**Fig supplementary captions**

**Fig. S1.** Multivariate statistical analysis of the metabolomic profiles between the RCBMC and CK cultivars.

**Fig. S2.** Heatmap of gene expression correlations among 10 sample groups.

**Fig. S3.** Zeatin biosynthesis pathway.

**Fig. S4.** Conserved domain of candidate UGTs

**Fig. S5.** Multiple sequence alignment of candidate UGTs protein sequences.

**Fig. S6**. Phylogenetic relationships and conserved motif compositions of candidate UGTs.

**Fig. S7.** Key parameter determination and module identification in Weighted Gene Co-expression Network Analysis (WGCNA).

**Fig. S8.** Module-trait relationship.

**Fig. S9.** FPKM values of the 14 screened transcription factors in RCBMC and CK.

**Fig. S10.** qPCR validation of differentially expressed genes.

.

**Fig. S1.** Multivariate statistical analysis of the metabolomic profiles between the RCBMC and CK cultivars. (a) OPLS-DA results for the RCBMC and CK cultivars; (b) OPLS-DA permutation test results for differential comparison groups.

**Fig. S2.** Heatmap of gene expression correlations among 10 sample groups.

**Fig. S3.** Zeatin biosynthesis pathway.

**Fig. S4.** Conserved domain of candidate UGTs

**Fig. S5.** Multiple sequence alignment of candidate UGTs protein sequences. The items marked with red borders are PSPG box sequences.

**Fig. S6**. Phylogenetic relationships and conserved motif compositions of candidate UGTs. The figure on the left shows a phylogenetic tree constructed from candidate genes and previously reported UGTs. Black nodes represent the screened candidate genes, red nodes represent known *O*-glycosyltransferases, and blue nodes represent known *C*-glycosyltransferases. The right panel shows the distribution of conserved motifs within these UGT proteins, as identified by the MEME suite. The ten uniquely identified motifs are represented by boxes of different colors. The black lines represent the non-conserved regions. The scale bar at the bottom indicates the length of the amino acid sequences.

**Fig. S7.** Key parameter determination and module identification in Weighted Gene Co-expression Network Analysis (WGCNA). (a) Determining the soft threshold. (b) Clustering dendrogram of genes.

**Fig. S8.** Module-trait relationship. The x-axis represents flavonoid metabolites, while the y-axis represents gene modules. Each cell contains the corresponding correlation coefficient and *P*-value, with the *P*-value indicated in parentheses.

**Fig. S9.** FPKM values of the 14 screened transcription factors in RCBMC and CK. Asterisks indicate a highly significant difference between the two groups based on Student’s t-test, **: p < 0.01, ***: p <0.001, ****: p <0.0001.

**Fig. S10.** qPCR validation of differentially expressed genes.
